# Institutional Housekeeping

**Published:** 1912-10-12

**Authors:** 


### INSTITUTIONAL HOUSEKEEPING.

### The Efficient Cook—From the Matron's Point of View.

It has been stated that the institution cook if efficient is never cheap. She requires good wages or she will be merely a bird of passage. Her wages will vary according to her appointments and the extent of her duties. The present average pay of a hospital cook, roughly speaking, is from about £2 10s. to £4 a month, according to the size of the institution. But, when all is taken into consideration, it will become a very acute problem whether she will not sometimes be in receipt of higher emoluments than what is accorded to her superior officer, the home or housekeeping sister. There can be no doubt that it is the lowness of salaries generally in hospital work which tends to make managers reluctant to pay enough to secure a well-trained and competent cook.

It is necessary, however, to take into consideration that neither sisters nor assistant matrons expect to remain in their posts for very long. They are acquiring experience which will stand them in good stead in their career, and hence they acquiesce in the fate which apportions to their labours a salary below market value.

The efficient cook should be a permanency, and every effort to retain her when once found is to the advantage of the institution. It is, perhaps, the best plan to give her the necessary pecuniary interest in her work by adopting the co-operative system of payment by results. This plan has been found in practice very successful. A certain preliminary wage is guaranteed to the cook on her engagement with an intimation that the rate at which her pay will increase depends entirely on herself.

As expenses are kept down, a high standard of quality, needless to say, being maintained, so her pay will increase. Under this arrangement it becomes her interest to economise in the true sense of the word. There is no doubt whatever that the economy of an establishment lies in the hands of the cook, and too much stress cannot be laid on this fact. However excellent the housekeeper may be, she cannot check the little daily leakages which mount up to such large dimensions in twelve months. The cook can, but cooks, however efficient, are but human. If she find at the end of the year that her constant care and attention mean so much more to her savings-bank book, it will mean that the institution has also been a gainer, and on a far larger scale. It is good business, to say the least of it, to pay ten or twenty pounds to gain hundreds.

Added to the monetary saving there is the inestimable value of personal interest in the particular institution to which she belongs. To the keen worker it means much to feel that she is an acknowledged factor in the well-being of her hospital. This spirit, always developed in the nursing staff, is seldom felt in the kitchen. But once evoked it will encourage the cook, as nothing else can, to stick to her work and bring it up to a state of efficiency. By adopting this system of co-operation a better class of woman could be induced to take up this branch of work, and it is only fair that if by her efforts she adds considerably to the cash balance of her hospital the cook should have full credit.

The question of how to obtain her is an important one to the matron. There are three good ways: (1) Through other members of the staff; (2) through a reliable registry office; (3) through the advertising medium of a good daily paper. The most satisfactory kind to engage is one who has been head cook in a smaller hospital or first assistant cook in a similar-sized hospital. It is advisable to have a personal interview always, for it is a regrettable fact that very few references are to be depended on. The engaging of a cook direct from a training school without practical experience in kitchen management is not advisable; for hospitals, both general and mental, require an entirely different method of management to any other kind of institution.

There is a fine field open to lady cooks in small hospitals and institutions. But in large hospitals and institutions where the kitchen department is under the direct control of the home sister or housekeeper the lady cook finds herself in the unenviable position of having all the responsibility and none of the authority. All who have worked in Government hospitals and institutions know what that means. It weakens her control over the rest of the staff and leaves her at the mercy of its plausible and unscrupulous members. Where the trained lady cook has entire charge of the kitchen arrangements and catering, with full authority over her staff, under the matron, she has proved herself to be the right woman in the right place.

### Housekeeping Queries.

Recipes for Fish Disguised in Flavour.

L. S., Plymouth.—*Fish Pudding* would suit perhaps. Take sufficient flesh from raw haddock, cod, or other white fish to weigh one pound, removing bones and skin. Chop it finely and mix with it 2 oz. of fresh white crumbs and 3 oz. of chopped suet. Pound these well. Add two teaspoonfuls of chopped parsley, two well-beaten eggs, one gill of milk or fish liquor, and seasoning. Mix thoroughly. Grease a mould or basin, press the mixture into it, twist a piece of greased paper over the top and steam for one hour. Turn out carefully and pour over egg or parsley sauce. *Baked Fish Soufflé* is popular. Melt 2 oz. of butter in a saucepan, add to it one teacupful of cooked mashed potato. Beat until hot and light, add one breakfastcupful of any cooked chopped fish, two tablespoonfuls of milk, two teaspoonfuls of chopped parsley, seasoning, and the yolks of two raw eggs. Lastly, stir in two whites of eggs stiffly whisked. Turn the mixture into a greased pie-dish, bake in a quick oven until puffed up, and brown and serve it in the dish at once.

NOTICE TO OUR READERS.

The Editor welcomes for Institutional Housekeeping any contribution from those engaged in the work of the commissariat in institutions. All accepted contributions are paid for. Queries answered on any subject relating to the supply, cost, storage, distribution, or preparation of food.

